# Comparing ChatGPT and GPT-4 performance in USMLE soft skill assessments

**DOI:** 10.1038/s41598-023-43436-9

**Published:** 2023-10-01

**Authors:** Dana Brin, Vera Sorin, Akhil Vaid, Ali Soroush, Benjamin S. Glicksberg, Alexander W. Charney, Girish Nadkarni, Eyal Klang

**Affiliations:** 1 Department of Diagnostic Imaging, Chaim Sheba Medical Center, Ramat Gan, Israel; 2 Faculty of Medicine, Tel-Aviv University, Tel-Aviv, Israel; 3 The Charles Bronfman Institute of Personalized Medicine, Icahn School of Medicine at Mount Sinai, New York, NY USA; 4 Division of Data-Driven and Digital Medicine (D3M), Icahn School of Medicine at Mount Sinai, New York, NY USA; 5 Hasso Plattner Institute for Digital Health, Icahn School of Medicine at Mount Sinai, New York, NY USA; 6 Division of Data-Driven and Digital Medicine (D3M), The Charles Bronfman Institute of Personalized Medicine, Icahn School of Medicine at Mount Sinai, New York, NY USA

**Keywords:** Medical ethics, Health care

## Abstract

The United States Medical Licensing Examination (USMLE) has been a subject of performance study for artificial intelligence (AI) models. However, their performance on questions involving USMLE soft skills remains unexplored. This study aimed to evaluate ChatGPT and GPT-4 on USMLE questions involving communication skills, ethics, empathy, and professionalism. We used 80 USMLE-style questions involving soft skills, taken from the USMLE website and the AMBOSS question bank. A follow-up query was used to assess the models’ consistency. The performance of the AI models was compared to that of previous AMBOSS users. GPT-4 outperformed ChatGPT, correctly answering 90% compared to ChatGPT’s 62.5%. GPT-4 showed more confidence, not revising any responses, while ChatGPT modified its original answers 82.5% of the time. The performance of GPT-4 was higher than that of AMBOSS's past users. Both AI models, notably GPT-4, showed capacity for empathy, indicating AI's potential to meet the complex interpersonal, ethical, and professional demands intrinsic to the practice of medicine.

## Introduction

Artificial intelligence (AI) is revolutionizing patient care and medical research. AI algorithms are being rapidly introduced and integrated into medical practice, enhancing human capacity and efficiency. Large language models (LLMs), specifically ChatGPT and GPT-4, have gained large scientific attention, with an increasing number of publications evaluating these models’ performance in medicine^1–6^. LLMs have proven proficient in diverse tasks, but their performance in areas requiring empathy and human judgment remains relatively unexplored.

The United States Medical Licensing Examination (USMLE) is a pivotal series of examinations that assess a wide range of skills indispensable for the practice of medicine. It not only measures a candidate's cognitive acuity and medical knowledge but also their ability to navigate complex interpersonal and system-based scenarios, uphold patient safety, and exercise professional legal and ethical judgments^7^. These ‘soft skills’ are critical to the effective and empathetic practice of medicine, enabling physicians to resonate with their patients and contribute positively to the healthcare system. Until March 2020, the Step2 Clinical Skills (CS) exam was the standard for assessing communication and interpersonal skills^8^. However, its suspension due to the COVID-19 pandemic underscored the need for an effective validation of these vital skills^9–11^. While the pandemic led to the discontinuation of the Step2 CS, its core elements of clinical communication are now integrated into the other Steps^12^, and the USMLE remains, for now, the official benchmark for assessing physicians’ communication abilities. Notably, USMLE Step2CK scores have been found to be predictive of performance across various residency performance domains, including patient care, teamwork, professionalism and communication^13^.

Artificial cognitive empathy, or AI’s ability to mimic human empathy, is an emerging area of interest. Accurate perception and response to patients’ emotional states are vital in effective healthcare delivery. Understanding AI’s capacity for this is particularly relevant in telemedicine and patient-centered care. Thus, the aim of this study was to evaluate the performance of ChatGPT and GPT-4 in responding to USMLE-style questions that test empathy, human judgment, and other soft skills.

## Methods

### Large language models

In this study, we assessed the performance of the AI models ChatGPT (GPT-3.5-turbo) and GPT-4, developed by OpenAI (https://openai.com). As language models, they are designed to process and generate text that mirrors human-like conversation. They achieve this by predicting what next word (token) should come next given an input, allowing them to generate coherent, contextually relevant responses. ChatGPT, an earlier version, has been widely used in a variety of applications due to its ability to generate coherent and contextually relevant responses. GPT-4, a newer paid version, is similar in design but much larger in scale, meaning it has been trained on more data and can understand and generate text with even greater accuracy.

### Medical questions datasets

To assess ChatGPT and GPT-4 we used a set of 80 multiple-choice questions designed to mirror the requirements of the USMLE examinations. This question set was compiled from two reputable sources. The first is a set of sample test questions for Step1, Step2CK, and Step3, released between June 2022 and March 2023, available at the official USMLE website (https://www.usmle.org/prepare-your-exam).

We screened all example test questions and selected 21 questions that did not require scientific medical knowledge, but required communication and interpersonal skills, professionalism, legal and ethical issues, cultural competence, organizational behavior, and leadership.

The second source is AMBOSS, a widely recognized question bank for medical practitioners and students (https://www.amboss.com/us), from which we selected an additional 59 questions. The chosen questions include Step1, Step2CK, and Step3-type questions, dealing with ethical scenarios, comparable to the questions chosen from the USMLE sample test questions. AMBOSS provides performance statistics from its past users, allowing a comparative analysis of LLMs’ performance against that of medical students and physicians.

Both ChatGPT and GPT-4 were tasked with responding to all the 80 questions included in this study, and their responses and performance were subsequently compared.

### Prompting

In line with standard methodological practices for assessing AI language models, we formatted a prompt structure which included the question text followed by the multiple-choice answers separated by a new line. Following the model’s response, we asked a follow-up question “Are you sure?”, which allowed us to evaluate the consistency and stability of the model and to elicit potential reevaluation of its initial response. If a model changes its answer, it may indicate that it possesses some level of 'uncertainty' about its initial response. Tracking how often and under what circumstances the model changes its answer can provide valuable insights into the model's capacity for self-revision, which is an important aspect of learning and decision-making systems. Prompt examples are shown in Supplementary items 1–4.

## Results

### Overall performance

**Figure 1:**
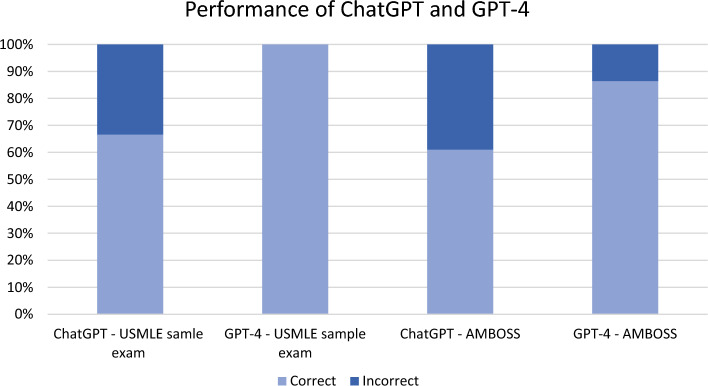
Performance of ChatGPT and GPT-4 on USMLE sample exam and AMBOSS questions.

In this study, a total of 80 multiple-choice USMLE soft-skills questions were included, and the different subjects are detailed in Table 1 and Fig. 1. ChatGPT accuracy for USMLE sample test and AMBOSS questions was 66.6% and 61%, respectively, with an overall 62.5% accuracy. GPT-4 demonstrated superior performance, with an accuracy of 100% and 86.4% for USMLE sample test and AMBOSS questions, respectively, and an overall accuracy of 90%. The results are also shown in Table 1 and in Fig. 1.

**Table 1:** Accuracy of ChatGPT and GPT4 on USMLE-type soft skills questions.

LLM	USMLE sample exam (n = 21), n (%)	AMBOSS (n = 59), n (%)	Overall (n = 80), n (%)
ChatGPT	14 (66.6)	36 (61)	50 (62.5)
GPT-4	21 (100)	51 (86.4)	72 (90)

### Consistency

Table 2 shows ChatGPT and GPT-4’s responses to the follow-up question “are you sure?”, when the initial answer was either correct or incorrect. GPT-4 exhibited 0% change rate when offered the opportunity to revise its responses, regardless of whether the initial response was correct or incorrect. In contrast, ChatGPT demonstrated a significantly higher rate of self-revision, altering its original answers 82.5% of the time when given the chance. When ChatGPT revised incorrect original responses, it was found that the model rectified the initial error and produced the correct answer in 53.8% of those cases. The results are detailed in Table 2.

**Table 2:** Consistency of ChatGPT and GPT-4.

LLM	Response to “Are you sure?”	Original answer Correct n (%)	Original answer Incorrect n (%)	Overall (n = 80), n (%)
ChatGPT		(n = 50)	(n = 30)	
	Change	40 (80)	26 (86.7)	66 (82.5)
	To correct	–	14 (53.8)	
	To incorrect	40 (100)	12 (46.2)	
	No Change	10 (20)	4 (13.3)	14 (17.5)
GPT-4		(n = 72)	(n = 8)	
	Change	0 (0)	0 (0)	0 (0)
	No Change	72 (100)	8 (100)	80 (100)

### LLM versus human

When comparing AI performance to human performance, AMBOSS’s user statistics reported an average correct response rate of 78% by its users for the same questions we utilized in our study. ChatGPT showed lower accuracy than human users, 61%, while GPT-4 had a higher accuracy rate of 86.4%.

## Discussion

This study evaluated ChatGPT and GPT-4 performance on USMLE multiple-choice questions assessing soft-skills such as empathy, ethics and judgment. There are several key findings. Both ChatGPT and GPT-4 answered correctly in the majority of the questions. GPT-4 outperformed ChatGPT, answering 90% of questions correctly compared to ChatGPT’s 62.5%. Additionally, GPT-4 demonstrated complete confidence in its responses, unlike ChatGPT, which displayed confidence in 17.5% of its answers.

Previous studies have demonstrated the ability of LLMs such as ChatGPT and GPT-4 to successfully pass the USMLE, with significantly better performance of GPT4. ChatGPT was shown to have 41–65% accuracy across Step1, Step2CK, and Step3 questions^14,15^, whereas GPT4 had an average score of 86%^16^. These previous studies evaluated the AI models across a spectrum of USMLE questions without specific focus on topics or distinguishing between medical knowledge-based queries and soft skill questions.

In our study, we focused on questions that require “human” qualities like empathy, communication, professionalism, and ethical judgment. We showed that LLMs demonstrate impressive results in these questions that test soft skills required from physicians. The superiority of GPT-4 was further confirmed in our study, with the model correctly answering 90% of the soft skill questions, in contrast to ChatGPT's accuracy of 62.5%. These results indicate that GPT-4 displays a greater capacity to effectively tackle not just medical knowledge-based questions but also those requiring a degree of empathy, ethical judgment, and professionalism.

Regarding the specific question datasets used in our study, a previous study that tested the performance of ChatGPT with a subset of 200 Step1 and Step2CK questions from AMBOSS reported accuracy of 44% and 42% respectively^14^. These are lower accuracy rates than the 61% accuracy ChatGPT demonstrated in our study. The difference may arise from the use of different questions from AMBOSS’s large database, but might also be because we focused on specific type of questions and did not include the whole span of topics covered in the USMLE.

The same study also tested ChatGPT on USMLE example test questions, out of which we selected questions for our study, and found that ChatGPT was accurate in 64.4% for Step1 and 57.8% for Step2. Another study that tested ChatGPT on the USMLE example test questions reported accuracies up to 55.8%, 59.1% and 61.3% for Step1, 2CK and 3, respectively^15^. In our study, ChatGPT showed similar but slightly higher results, with accuracy of 66.6%.

Beyond question types, it's also noteworthy to address the behavioral tendencies of the two models. ChatGPT's inclination to adjust its initial responses may suggest a design emphasis on adaptability and flexibility, possibly favoring diverse interactions in conversational contexts. On the other hand, GPT-4's consistency could point to its robust training or a sampling mechanism predisposed to stability. This distinction is significant as it highlights the difference between a model's inherent adaptability for dynamic settings versus consistent output in more stable contexts.

When comparing AI performance to human performance, AMBOSS's user statistics provide an invaluable benchmark. AMBOSS reported an average correct response rate of 78% by its users for the same questions we utilized in our study. ChatGPT showed lower accuracy than human users, implying a shortage of relevant soft skills compared to medical students and doctors. GPT-4 surpassed this human performance metric, suggesting its capabilities in solving complicated ethical dilemmas, demonstrating empathy, and managing patients and their families in a professional manner that physicians are required to exhibit.

The potential of AI to display empathetic responses is a topic of increasing interest. A notable recent study compared responses from ChatGPT and physicians to patient inquiries on a social media platform and found that ChatGPT's responses were viewed as more empathetic, emphasizing AI's potential to emulate human-like empathy^17^.

There are several limitations to our study. First, the question pool used in this study was limited, including only 80 multiple-choice questions from two different sources, with potential introduction of selection bias. These may not accurately reflect the actual USMLE questions and may not encompass all aspects of 'soft skills' that are essential to medical practice. In addition, consistency levels of the two models were assessed based on an opportunity to revise their answers. However, this mechanism for potential reevaluation might not translate to human cognition understanding of ‘uncertainty’, as these models operate based on calculated probabilities for an output, rather than human-like confidence. This simplification potentially limits the depth of our understanding of the models' decision-making processes.

To conclude, our findings underscore the potential role of LLMs in augmenting human capacity in healthcare, especially in areas requiring empathy and judgment. GPT-4's performance surpassed the human performance benchmark, emphasizing its potential in handling complex ethical dilemmas that involve empathy, which are critical for patient management.

Future research should consider larger and more diverse question pools and ethical scenarios to better represent the full range of soft skills important to medical practice. Such studies would provide a more comprehensive understanding of LLMs' capabilities in these areas and explore the applicability of these LLMs in real-world clinical settings.

### Supplementary Information

Supplementary Information 1.

Supplementary Information 2.

## Data Availability

The data supporting the findings of this study are included as an appendix to this document. The dataset comprises a comprehensive spreadsheet containing all the questions and corresponding answers utilized in the research.
